# Enhancing the durable performance of LiMn_2_O_4_ at high-rate and elevated temperature by nickel-magnesium dual doping

**DOI:** 10.1038/s41598-019-53494-7

**Published:** 2019-11-14

**Authors:** Yue Yu, Junming Guo, Mingwu Xiang, Changwei Su, Xiaofang Liu, Hongli Bai, Wei Bai, Kaijiao Duan

**Affiliations:** 1 Key Laboratory of Green-chemistry Materials in University of Yunnan Province, Yunnan Minzu University, Kunming, 650500 China; 2 National and Local Joint Engineering Research Center for Green Preparation Technology of Biobased Materials, Yunnan Minzu University, Kunming, 650500 China

**Keywords:** Environmental social sciences, Composites

## Abstract

Various nickel and magnesium dual-doped LiNi_*x*_Mg_0.08_Mn_1.92−*x*_O_4_ (*x* ≤ 0.15) were synthesized via a modified solid-state combustion method. All as-prepared samples show typical spinel phase with a well-defined polyhedron morphology. The Ni-Mg dual-doping obviously decreases the lattice parameter that gives rise to the lattice contraction. Owing to the synergistic merits of metal ions co-doping, the optimized LiNi_0.03_Mg_0.08_Mn_1.89_O_4_ delivers high initial capacity of 115.9 and 92.9 mAh·g^−1^, whilst retains 77.1 and 69.7 mAh·g^−1^ after 1000 cycles at 1 C and high current rate of 20 C, respectively. Even at 10 C and 55 °C, the LiNi_0.03_Mg_0.08_Mn_1.89_O_4_ also has a discharge capacity of 92.2 mAh·g^−1^ and endures 500 cycles long-term life. Such excellent results are contributed to the fast Li^+^ diffusion and robust structure stability. The anatomical analysis of the 1000 long-cycled LiNi_0.03_Mg_0.08_Mn_1.89_O_4_ electrode further demonstrates the stable spinel structure via the mitigation of Jahn-Teller effect. Hence, the Ni-Mg co-doping can be a potential strategy to improve the high-rate capability and long cycle properties of cathode materials.

## Introduction

The lithium ion batteries (LIBs) are now have received extensive attention, mainly in electronic equipment and new energy vehicles. Among the cathode materials, spinel LiMn_2_O_4_ has received widespread attention for large-scale application due to the high abundance, nontoxicity, good thermal stability and high security^1–3^. However, LiMn_2_O_4_ suffers from sever capacity fading upon long electrochemical cycling at elevated temperature owing to the dissolution of manganese (Mn^3+^ → Mn^4+^ + Mn^2+^), Jahn-Teller effects, and so on^4,5^.

To address forementioned problem, surface coating technology and metal cationic doping are generally used as effective approaches to inhibit the Jahn-Teller distortions and stabilize the spinel crystal structure of LiMn_2_O_4_. Replacement of manganese ions with metal cations, such as Al^6^, Ni^7,8^, Cr^9^, Co^10^, Mg^11^ and Ce^12^, has been successfully used to minimize capacity fade. Among them, the average ionic radius of nickel (II) ions is 0.69 nm, which is similar to the Mn^3+^ ion (r = 0.65 nm) in crystalline LiMn_2_O_4_. Also, the bond energy of Ni-O is stronger than that of Mn-O bond. Yuan *et al*.^13^ showed that Ni^2+^ belongs to the 3d-metals and can replace the Mn^3+^ in LiMn_2_O_4_ structure, and the bond length of the Ni-O (0.1915 nm) is shorter than that of the Mn-O bond (0.1937 nm), hence strengthening the structural stability of spinel LiMn_2_O_4_^14^. Likewise, the previous other work^14–16^ also affirmed that moderate Ni-doping can limit the Jahn-Teller effects thereby stabilize the spinel structure of LiMn_2_O_4_. By contrast, Mg-doping is conductive to enhancing the cycling stability due to the fact that reduces the polarization and improves the kinetic properties via increasing the electronic conductivity^17^. Xiang *et al*. have reported a solid-state combustion method to synthesize the Mg-doped LiMn_2_O_4_ cathode materials, which delivered good cycle stability because of the Mg-doping reduces the Jahn-Teller effects^11^. Deng *et al*.^18^ also manifested that the Mg-doped LiMn_2_O_4_ cathode materials have enhanced cycling performance at elevated temperature. Moreover, elemental Mg is rich, non-toxic and inexpensive, especially lighter than other metal ions.

Based on above these advantages of Ni^2+^ and Mg^2+^ ions, Zhang *et al*.^19^ prepared the Ni-Mg co-doped LiMn_2_O_4_ cathode materials using microwave irradiation as a sintering technique. The resultant LiNi_0.03_Mg_0.02_Mn_1.95_O_4_ delivered a capacity of about 120.0 mAh·g^−1^ at 1 C and remained 90.6 mAh·g^−1^ at a high current rate of 10 C. Even at an elevated temperature of 55 °C, the LiNi_0.03_Mg_0.02_Mn_1.95_O_4_ also obtained the high capacity of 90.0 mAh·g^−1^ at a higher rate of 10 C. In addition to the Ni-Mg co-doping strategy, other metals such as Ni-Mo^20^, Mg-Si^21^, and Ni-Ti co-doping^22^ were also employed to improve the cycling property and structure stability of spinel LiMn_2_O_4_ cathode materials. For these dual-doped LiMn_2_O_4_ cathode materials, the traditional preparation methods including high-temperature solid-state reaction, sol-gel method, microwave irradiation and so on need long reaction time, high temperature and troublesome pre-treatment. Compared to high-temperature solid-state reaction, the solid-state combustion method has the advantages of time-saving and energy efficient and avoids oxygen deficiency. Additionally, different synthesis method lead spinel LiMn_2_O_4_ have unique various morphology, so indicating various electrochemical performance. The high-rate capability and durable cycling performance is closely related to its kinetic properties, such as lithium ion diffusion coefficient and activation energy. Therefore, introducing an feasible method to synthesize the LiNi_*x*_Mg_0.08_Mn_1.92−*x*_O_4_ cathode materials facilely is a great challenge, whilst considerable attention should focus on structure, morphology and detailed high-rate and durable elevated temperature properties.

In this work, Ni-Mg co-doped LiNi_*x*_Mg_0.08_Mn_1.92−*x*_O_4_ (*x* ≤ 0.15) cathodes with polyhedron morphology were prepared by a facile solid-state combustion method. The effects of the Ni-Mg co-doping on the structure, morphology, high-rate and long cycle performance as well as kinetic properties of the LiNi_*x*_Mg_0.08_Mn_1.92−*x*_O_4_ cathode materials were investigated detailedly. Furthermore, the structure characterization of long-cycled electrode materials was performed to further determine the stability and Li-ion kinetics. The resultant optimal Ni-Mg co-doped LiMn_2_O_4_ sample presented excellent high-rate capability, long cycling stability and high temperature performance.

## Experiment Section

### Preparation of materials

A series of LiNi_*x*_Mg_0.08_Mn_1.92−*x*_O_4_ (*x* ≤ 0.15) products were synthesized by the solid-state combustion method using citric acid as a fuel. Firstly, with a total mass of reaction mixture is 6.0 g, the lithium carbonate (AR, Aladin), manganese carbonate (AR, Aladin), nickel acetate and magnesium acetate (AR, Aladin) were weighed according to the stoichiometric ratio of 1:(1.92 − *x*):*x* :0.08 (Li:Mn:Ni:Mg). Then adding 0.3 g citric acid into a polytetrafluoroethylene jar and using the ethanol as medium. Secondly, the mixture was ball-milled for 10 h by planetary. Thirdly, the mixture was dried at 80 °C in an oven. Thirdly, the as-obtained powder was calcined in a muffle furnace at 500 °C for 1 h. The pre-product was obtained after naturally cooling. Immediately, the pre-product was calcined again at 650 °C for 6 h, then cooled to room temperature and ground to obtain the ultimate LiNi_*x*_Mg_0.08_Mn_1.92−*x*_O_4_ (*x* ≦ 0.15) cathode materials.

### Materials characterization

The crystalline phase of the samples was identified by powder X-ray diffraction (XRD, Bruker Company) using Cu Kα radiation (λ = 0.15406 nm) over the 2θ range of 10°–70°. Morphological and particle size was examined by scanning electron microscopy (SEM, QUANTA-200 America FEI Company) and transmission electron microscopy (TEM, JEM-2100, Japan Electronics Corporation). X-ray photoelectron spectroscopy (XPS, Thermo fisher Scientific) analysis was performed by using Al Kα (1486.6 eV) radiation. The cycled electrodes were disassembled, washed with NMP and dried, further characterized by the XRD, SEM and TEM tests.

### Cell assemble and electrochemical measurement

The electrochemical performance of as-synthesized LiNi_x_Mg_0.08_Mn_1.92−x_O_4_ samples was evaluated in CR2032 type coin cells using lithium metal as the anode and reference electrode. The working electrodes were fabricated by mixing active materials, carbon black and polyvinylidene fluoride (PVDF) binder in 1-methyl-3-pyrrolidone (NMP) solvent with a mass ratio of 8:1:1. The electrolyte was 1 M LiPF_6_ that dissolved in ethylene carbonate (EC), dimethyl carbonate (DMC) and methyl ethyl carbonate (EMC) at a volume ratio 1:1:1. The electrochemical cells were assembled in a high-purity argon atmosphere (<1 ppm of O_2_ and H_2_O). The electrode activities were performed at various current rate (1 C is defined as 148.0 mAh·g^−1^) and voltage range from 3.0 to 4.5 V (vs. Li^+^/Li) by using Land CT2001A system (Wuhan Jinnuo Electronics). The cyclic voltammogram (CV) measurements at a scan rate of 0.05 mV·s^−1^ and the electrochemical impedance spectroscopy (EIS) tests in the frequency range of 0.1 Hz to 100 kHz were performed on an electrochemical workstation (Shanghai Chenhua Instrument Co., Ltd.).

## Results and Discussions

**Figure 1:**
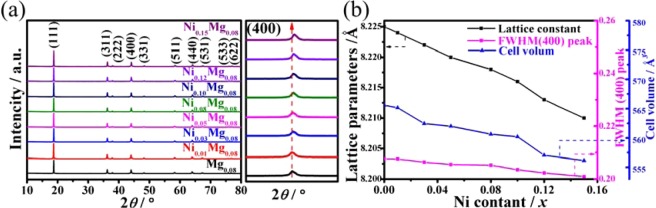
(**a**) XRD patterns of LiNi_*x*_Mg_0.08_Mn_1.92−*x*_O_4_ samples, the amplified pattern of (400) peak, (**b**) the lattice parameters, FWHM (400) peak and cell volume of LiNi_*x*_Mg_0.08_Mn_1.92−*x*_O_4_ samples.

Figure 1(a) exhibits the XRD patterns of the LiNi_*x*_Mg_0.08_Mn_1.92−*x*_O_4_ (x ≤ 0.15) materials. All the diffraction peaks with high crystallinity corresponded to the cubic spinel LiMn_2_O_4_ (JCPDS No. 35-0782), showing that the Ni-Mg co-doping doesn’t change the pristine spinel structure. The amplified pattern of (400) peaks present a slight movement towards the larger angle for the Ni-Mg co-doped samples, which indirectly interprets the decrease of the unit cell volume of the co-doped samples (Fig. 1b). As shown in Fig. 1(b), the lattice parameters of the LiNi_*x*_Mg_0.08_Mn_1.92−*x*_O_4_ samples display the decrease trend with the increased Ni^2+^ content. Generally, the Mn^4+^ exhibits an ionic radius of 0.53 Å, while the Mn^3+^ shows two ionic radius of 0.58 Å and 0.645 Å in low spin state and high spin state, respectively^23^. In this regard, the high-spin state Mn^3+^ ions (0.645 Å) is considered to be substituted due to the similar ionic radius of Ni^2+^ (r = 0.69 Å) and Mg^2+^ (0.65 Å), to balance the valence electrons in this structure, the low-spin state trivalent manganese ion would change to tetravalent manganese ion. The above two reasons lead to the decreased lattice constant in the doped samples. It has been confirmed that the Ni-Mg co-doping is attributed to the cell volume contraction, which is due to the the Ni-O bond (0.1915 nm) is shorter than that the Mn-O bond (0.1937 nm), and the of the Mg-O bonding energy is stronger than the Mn-O boding energy^14^. Moreover, the FWHM of (400) peaks gradually decreased with the increasing of Ni^2+^ amount, which demonstrate the improved crystalline quality.

**Figure 2:**
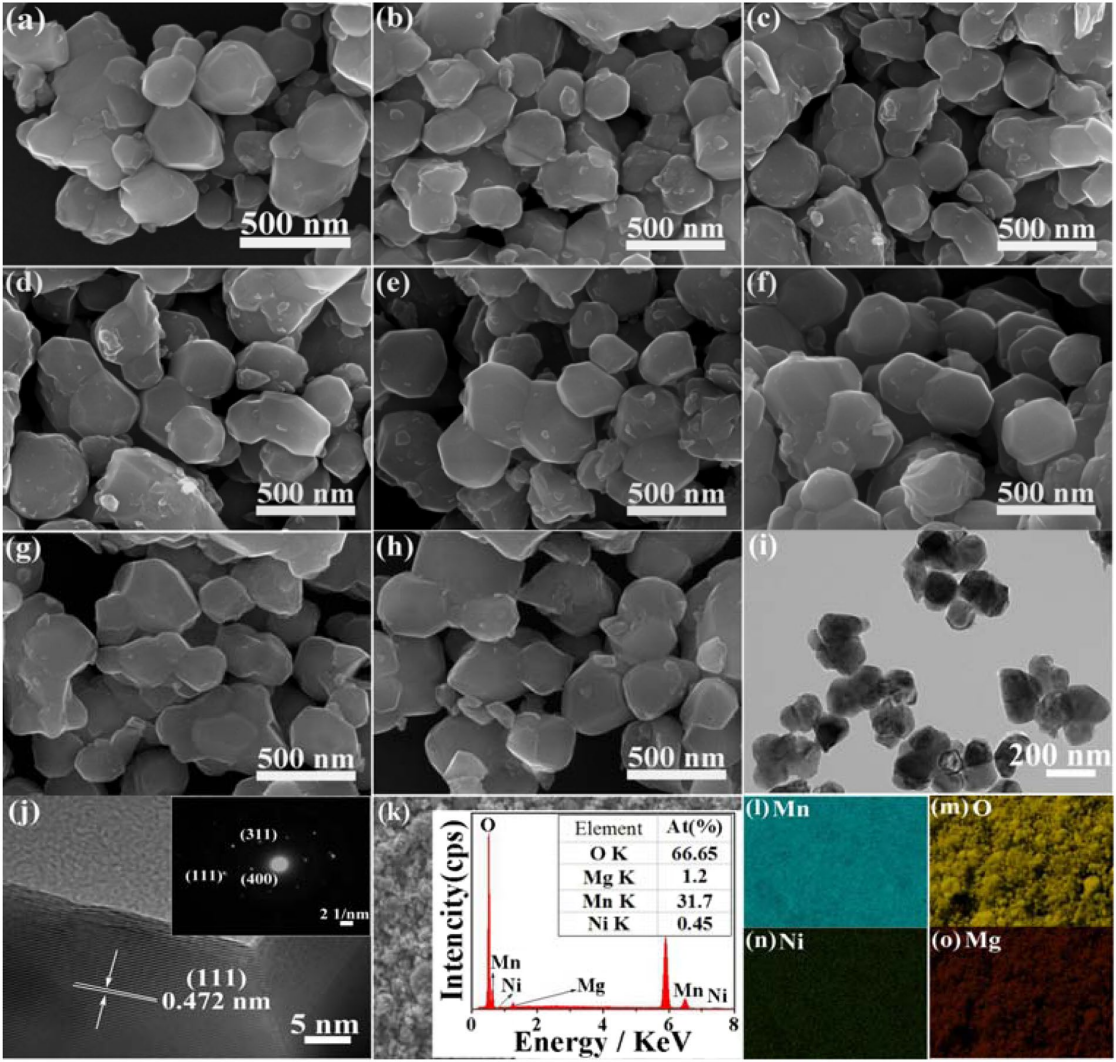
SEM images of LiNi_*x*_Mg_0.08_Mn_1.92−*x*_O_4_ samples (**a**) *x* = 0, (**b**) *x* = 0.01, (**c**) *x* = 0.03, (**d**) *x* = 0.05, (**e**) *x* = 0.08, (**f**) *x* = 0.10, (**g**) *x* = 0.12 and (**h**) *x* = 0.15, (**i**) TEM and (**j**) high-resolution transmission electron microscopy (HRTEM) images of LiNi_0.03_Mg_0.08_Mn_1.89_O_4_ sample, the inset is the corresponding selected area electron diffraction (SAED) pattern, (**k**–**o**) mapping analysis for Mn, O, Ni and Mg in the LiNi_0.03_Mg_0.08_Mn_1.89_O_4_ sample.

Figure 2(a–h) is the SEM images of various LiNi_*x*_Mg_0.08_Mn_1.92−*x*_O_4_ (*x* ≤ 0.15) samples. All the materials have the well-defined polyhedron morphology with the particle size of 150-250 nm, whilst exhibit slightly agglomeration. On the basis of comparative results, different Ni-doping contents (when *x* ≤ 0.15) have no significant effect on the micromorphology of the Ni-Mg co-doping samples. Figure 2(i) displays the TEM image of the LiNi_0.03_Mg_0.08_Mn_1.89_O_4_ sample and further confirm the polyhedral morphology. The HRTEM image of the LiNi_0.03_Mg_0.08_Mn_1.89_O_4_ sample is also provided in Fig. 2(j). As seen, the The lattice fringe of (111) plane is 0.472 nm and these diffraction spots (inset in Fig. 2j) are indexed to the planes of (311), (400) and (111) of the cubic LiMn_2_O_4_ structure. To demonstrate the elemental composition of the Ni-Mg dual-doped samples, Fig. 2(k–o) shows the EDS mapping of the LiNi_0.03_Mg_0.08_Mn_1.89_O_4_. The specific content (at.%) of O, Mg, Mn and Ni is approximately closed to the theoretical atomic ratio. Furthermore, these mapping results elucidate the presence of Mn, O, Ni and Mg elements, which are uniformly distributed in the LiNi_0.03_Mg_0.08_Mn_1.89_O_4_.

**Figure 3:**
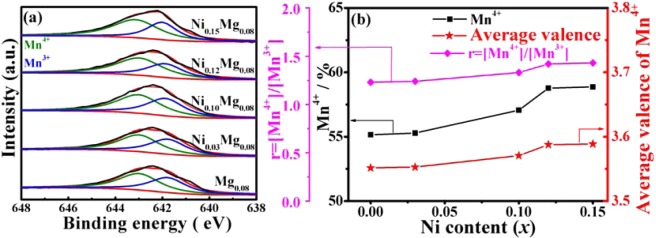
(**a**) XPS spectra of Mn2p_3/2_ peaks and (**b**) the cation distribution of Mn and r value in the LiNi_*x*_Mg_0.08_Mn_1.92−*x*_O_4_ sample.

The surface chemical compositions and distribution of the as-prepared materials were further determined by XPS, as shown in Fig. 3(a). The Mn2p_3/2_ binding energy is about at 642.5 eV, demonstrating that the Mn valance state in the co-doped spinel LiMn_2_O_4_ is the Mn^3+^ and Mn^4+^. On the basis of the XPS data, Fig. 3(b) shows the cation distribution of Mn in LiNi_*x*_Mg_0.08_Mn_1.92−*x*_O_4_ samples. Noted that the content of Mn^4+^, the r value of [Mn^4+^]/[Mn^3+^] and the average oxidation state of manganese show a gradually increasing trend with the enhanced Ni^2+^ content, and when the Ni^2+^ content up to 0.15, these tendencies are nearly stable. As Ding *et al*. reported^24^ that the lattice structure stability is depending on the ratio r = [Mn^4+^]/[Mn^3+^], Jahn-Teller transition becomes smaller when r ≥ 1.18. Seen in the Fig. 3(b), the r values of all samples are bigger than 1.18, which demonstrates that the Ni-Mg co-doping can actively limit Jahn-Teller effects of the LiMn_2_O_4_. In addition, the increase in the Mn^4+^ content and the average oxidation state of Mn can enhance the structure stability and reduced the dissolution of Mn, hence improving the high rate capacity of the LiMn_2_O_4_ cathodes.

**Figure 4:**
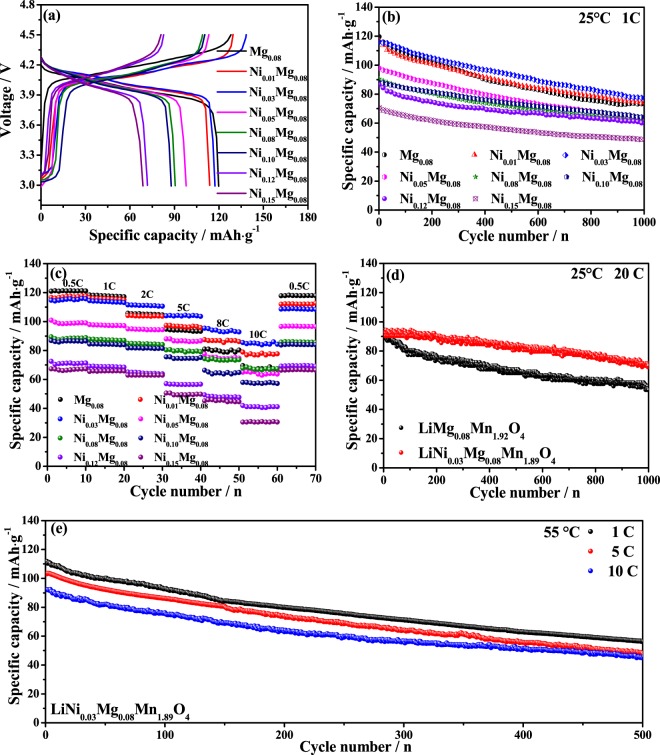
Electrochemical performances of LiNi_*x*_Mg_0.08_Mn_1.92−*x*_O_4_ samples: (**a**) initial charge-discharge curves, (**b**) cyclic performances at 1 C, and (**c**) rate capability at 25 °C, (**d**) the cyclic performances of LiMg_0.08_Mn_1.92_O_4_ and LiNi_0.03_Mg_0.08_Mn_1.89_O_4_ samples at 20 C and (**e**) the cyclic performances of LiNi_0.03_Mg_0.08_Mn_1.89_O_4_ sample at various current rate and 55 °C.

Figure 4(a) depicts the initial charge/discharge curves of the LiNi_*x*_Mg_0.08_Mn_1.92−*x*_O_4_ samples at 1 C between of 3.0 and 4.5 V at 25 °C. Seen that two well-defined voltage plateaus at 3.9–4.3 V can be observed for all samples, corresponding to a representative two-step intercalation/de-intercalation process of LiMn_2_O_4_. With the increased Ni^2+^ content from x = 0 to 0.03, the charge voltage platforms are gradually descend, whilst the discharge platforms are elevated, however, when the Ni^2+^ content increases from *x* = 0.05 to 0.15, the opposite can be true, which implying that the polarization of the electrode is increased. Figure 4(b) is the corresponding cycling performance of the LiNi_*x*_Mg_0.08_Mn_1.92−*x*_O_4_ (*x* ≤ 0.15) samples at 1 C and 25 °C. As shown, the cycling stability of the Ni-Mg dual-doped materials firstly rise with the increased Ni^2+^ content from *x* = 0 to 0.03. When the Ni^2+^ content gradually increases, the capacity decreases by degrees. Moreover, the introduction of Ni^2+^ and Mg^2+^ in the spinel structure weakens the first capability to some extent. This unfortunate results is attributed to the reduction of electrochemically active trivalent manganese ions. According to the previous reports^25^, the traditional Li^+^ diffusion in the spinel structure is along the zigzag that hop from the 8a position to 16c site, providing that the next 8a site is vacant. When the Ni^2+^ content increases, the 8a site will be replaced by increased Ni^2+^ ions, so the Li^+^ diffusion pathway is blocked by Ni^2+^ ions. As a result, the initial capacity of the materials is greatly reduced when the nickel ions are excessive. Among all samples, the optimal LiNi_0.03_Mg_0.08_Mn_1.89_O_4_ presents a initial capacity of 115.9 mAh·g^−1^ with an excellent capacity retention of 67% after 1,000 cycles. Figure 4(c) shows the discharge capacities cycled sequentially from 0.5 C to 10 C. As shown, the discharge specific capacities of all samples show a downward trend as the increased discharge rate. This is mainly because that the de-intercalation/intercalation process of Li^+^ ions is hindered at the high rate^26^. Noted that the LiNi_0.03_Mg_0.08_Mn_1.89_O_4_ exhibited a good rate performance than other samples at higher rate, which is attributed to the addition of Mg^2+^ ions that enhance the ionic conductivity by lowering local Li^+^ ions reaction energy barrier barriers^25^.

Additionally, Fig. 4(d) also demonstrates the high-rate capability comparison of the LiMg_0.08_Mn_1.92_O_4_ and LiNi_0.03_Mg_0.08_Mn_1.89_O_4_ samples at the higher current rate of 20 C. Note that the initial discharge capacity of the LiNi_0.03_Mg_0.08_Mn_1.89_O_4_ is up to 92.9 mAh·g^−1^, even after 1,000 cycles, about 75% of its initial capacity can still be retained, whereas the LiMg_0.08_Mn_1.92_O_4_ is 56%. These results prove a stable spinel structure of the Ni-Mg co-doping LiNi_0.03_Mg_0.08_Mn_1.89_O_4_ at high current rate. To further evaluate the robust structure stability, the elevated-temperature cycling performance of the LiNi_0.03_Mg_0.08_Mn_1.89_O_4_ sample was performed at various current rate and 55 °C, as shown in Fig. 4(e). The discharge capacity of LiNi_0.03_Mg_0.08_Mn_1.89_O_4_ sample displays smaller downward trend as the discharge rate increases. The initial capacity is 106.8 mAh·g^−1^, 103.2 mAh·g^−1^ and 92.2 mAh·g^−1^ at 1, 5 and 10 C, respectively. Even at 10 C after 500 cycles, the 45.0 mAh·g^−1^ can be maintained. The above results demonstrate the improvement effect of Ni^2+^ and Mg^2+^ dual-doped on the discharge capacity at high rate and temperature.

**Figure 5:**
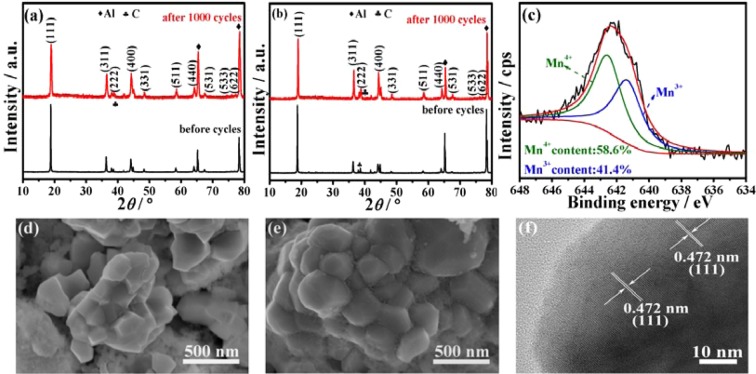
The analysis figures after 1,000 cycles of 1 C: XRD patterns of (**a**) LiMg_0.08_Mn_1.92_O_4_ and (**b**) LiNi_0.03_Mg_0.08_Mn_1.89_O_4_ samples, (**c**) XPS spectrum of Mn2p_3/2_ peaks in LiNi_0.03_Mg_0.08_Mn_1.89_O_4_, (**d**), (**e**) SEM images of LiMg_0.08_Mn_1.92_O_4_ and LiNi_0.03_Mg_0.08_Mn_1.89_O_4_, (**f**) HRTEM image of LiNi_0.03_Mg_0.08_Mn_1.89_O_4_.

To further study the structural stability of the materials, Fig. 5(a,b) shows the contrastive XRD patterns after 1,000 cycles at 1 C and 25 °C. The two electrodes have the similar diffraction patterns before cycle and after 1,000 cycles, indicating an integrated spinel structure of the LiMn_2_O_4_ with a Fd3m space group. Especially, compared with the cycled LiMg_0.08_Mn_1.92_O_4_ sample, the LiNi_0.03_Mg_0.08_Mn_1.89_O_4_ sample shows relatively higher peak intensity and narrower FWHM, implying that the co-doped cathode maintain a good crystallinity and enhanced cycling performance. The composition of the LiNi_0.03_Mg_0.08_Mn_1.89_O_4_ electrode after 1000 cycles was also further analyzed by XPS. The Mn2p_3/2_ spectra is shown in Fig. 5(c). The Mn2p_3/2_ spectrum contains two peaks—Mn^4+^ (in MnO_2_ or LiMn_2_O_4_) and Mn^3+^ (in Mn_2_O_3_ or LiMn_2_O_4_)^27^. The content of Mn^4+^ peak was determined after 1000 cycles to be greater than those of the fresh electrode (as shown in Fig. 3), indicating the Mn^3+^ ions have dissolved during the charge-discharge process, so the later discharge capacity is relatively lower.

The SEM images of the cycled LiMg_0.08_Mn_1.92_O_4_ and LiNi_0.03_Mg_0.08_Mn_1.89_O_4_ electrodes are provided to observe the effect of Ni and Mg co-doping on the stability of the spinel structure, as shown in Fig. 5(d,e). After 1,000 cycles of 1 C, both the two cathodes still maintain inherent polyhedral morphology like the fresh cathodes. No other significant structure transformation or particles damage is observed, except there is small amount of PVDF and carbon black on the particle surface. This result could explain that why the LiMg_0.08_Mn_1.92_O_4_ and LiNi_0.03_Mg_0.08_Mn_1.89_O_4_ cathodes deliver the similar electrochemical performance at low current rate of 1 C, as shown in the above Fig. 4b. In order to detect any structure modifications after the long-cycled electrochemical measurement, the LiNi_0.03_Mg_0.08_Mn_1.89_O_4_ sample was further detected using HRTEM. As shown in Fig. 5(f), the crystalline planes (111) of LiMn_2_O_4_ (JCPDS NO.35-0782) with corresponding distance of 0.472 nm can be confirmed, no other peaks were detected after 1000 cycles. This results are in accordance with the XRD analysis.

**Figure 6:**
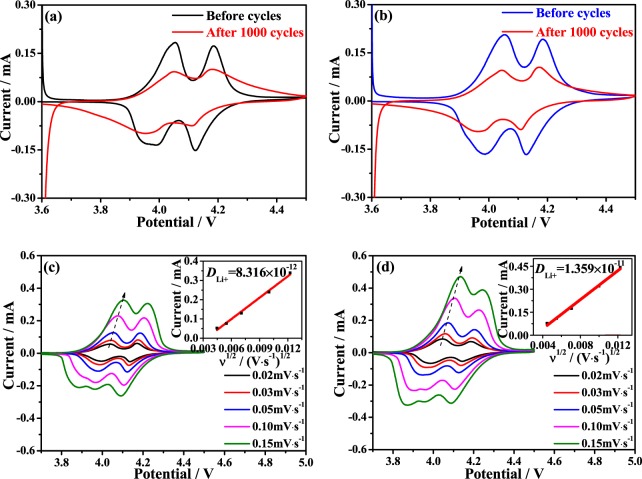
(**a**,**b**) Cyclic voltammetric curves of LiMg_0.08_Mn_1.92_O_4_ and LiNi_0.03_Mg_0.08_Mn_1.89_O_4_ samples in the potential range of 3.6-4.5 V (vs. Li^+^/Li) at a scan rate of 0.05 mV·s^−1^ before and after 1000 cycles, (**c**,**d**) cyclic voltammetric curves of LiMg_0.08_Mn_1.92_O_4_ and LiNi_0.03_Mg_0.08_Mn_1.89_O_4_ electrodes at different scan rates, the insets in c and d are the plots of peak current vs. square root of the scan rate.

Figure 6 shows the CV curves of LiMg_0.08_Mn_1.92_O_4_ and LiNi_0.03_Mg_0.08_Mn_1.89_O_4_ samples. For the LiMg_0.08_Mn_1.92_O_4_ sample, the two pairs of redox peaks shown in the Fig. 6(a) have two pairs of redox peaks corresponding to two-step de-intercalation/intercalation of Li^+^ ions. After 1000 cycles, peak currents are decreased significantly, indicating a relatively poor cycling stability of the LiMg_0.08_Mn_1.92_O_4_ sample. As shown in Fig. 6(b), the corresponding peak symmetry of the LiNi_0.03_Mg_0.08_Mn_1.89_O_4_ changed relatively little. These suggest that the addition of Ni^2+^ and Mg^2+^ can improve the reversibility of lithium ions.

The lithium ion diffusion coefficient (*D*_Li+_) can be calculated according to the following equation^28^:

1 $${{i}}_{{\rm{p}}}=({\rm{2.69}}\times {{\rm{10}}}^{{\rm{5}}}){n}^{{\rm{3}}/{\rm{2}}}{{C}}_{{\rm{Li}}+}{A}{({D}_{{\rm{Li}}+})}^{{\rm{1}}/{\rm{2}}}{{v}}^{{\rm{1}}/{\rm{2}}}(\mathrm{25}\,^\circ C)$$

where *i*_p_ is the value of peak current (mA), n is the electron transfer number (*n* ≈ 1 for spinel LiMn_2_O_4_), *C*_Li+_ is the bulk concentration of Li^+^ (given as 0.02378 mol·cm^−3^ for spinel LiMn_2_O_4_), *D*_Li+_ stands for the Li^+^ diffusion coefficient (cm^2^·s^−1^) and *v* represents the scan rate (mV·s^−1^). As seen from Fig. 6(c,d), the peak current increases with the increased scan rate, and the peak current and peak area of the LiMg_0.08_Mn_1.92_O_4_ are smaller than the LiNi_0.03_Mg_0.08_Mn_1.89_O_4_. According to the equation, the *D*_Li+_ is calculated as shown in the insets of Fig. 6(c,d). The *D*_Li+_ of the LiMg_0.08_Mn_1.92_O_4_ and LiNi_0.03_Mg_0.08_Mn_1.89_O_4_ is 8.316 × 10^−12^ and 1.359 × 10^−11^ cm^2^·s^−1^, respectively. Such a larger *D*_Li+_ in LiNi_0.03_Mg_0.08_Mn_1.89_O_4_ sample is in well accordance with the rate performance in Fig. 4(c), indicating the fast Li^+^ ions diffusion rate.

**Figure 7:**
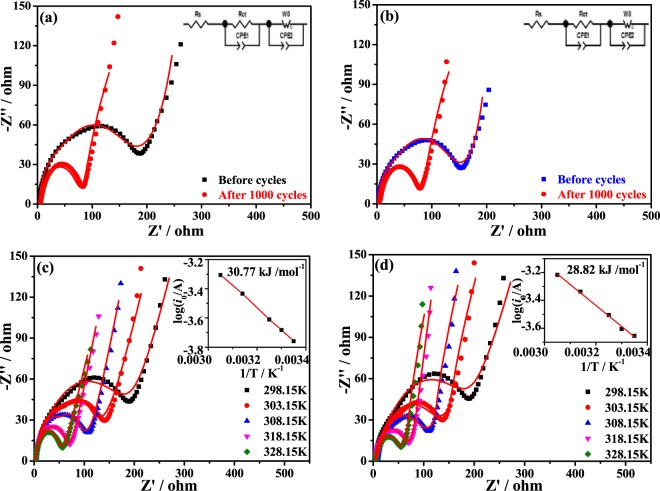
Nyquist plots of (**a**) LiMg_0.08_Mn_1.92_O_4_ and (**b**) LiNi_0.03_Mg_0.08_Mn_1.89_O_4_ before cycling and after 1000 cycles, the insets in a and b are the equivalent circuit, Nyquist plots of (**c**) LiMg_0.08_Mn_1.92_O_4_ and (**d**) LiNi_0.03_Mg_0.08_Mn_1.89_O_4_ electrodes at different temperatures, the insets in c and d are the Arrhenius plots of log (*i*_0_/A) vs. 1/*T*.

Figure 7(a,b) presents the Nyquist plots of LiMg_0.08_Mn_1.92_O_4_ and LiNi_0.03_Mg_0.08_Mn_1.89_O_4_ electrodes, respectively. An equivalent circuit model was used to fit the impedance signal (as seen the insets in Fig. 7a,b). This circuit included ohmic resistance of electrolyte (R_s_), charge transfer resistance (R_ct_), double layer capacitance (*CPE*), and Warburg impedance (*W*)^29^. The R_ct_ values of LiNi_0.03_Mg_0.08_Mn_1.89_O_4_ are 146.9 Ω and 64.9 Ω before and after 1000 cycles, respectively. By contrast, the LiMg_0.08_Mn_1.92_O_4_ presents the higher R_ct_ values of 177.6 Ω and 77.6 Ω, respectively. These results indicate a faster lithium ions diffusion rate in the Ni-Mg co-doped samples. To further explore the energy among the Li^+^ ions diffusion, the activation energy (*E*_a_) was tested by impedance method. Figure 7(c,d) shows the Nyquist plots for each electrode at different temperatures. So the activation energy will be calculated by the following equations:

2 $${i}_{0}=RT/nF{{\rm{R}}}_{{\rm{ct}}}$$

3 $${i}_{0}=A\,\exp \,(-\,{E}_{a}/RT)$$

where *i*_0_ stands for the exchange current, *R* represents the gas constant (8.314 J·mol^−1^·K^−1^), *T* (K) is the absolute temperature, n is the number of the electron transfer (*n* ≈ 1 for spinel LiMn_2_O_4_), *F* is the Faraday constant (96484.5 C·mol^−1^), *A* is a temperature coefficient. Combined with Eqs 2 and 3, the equation of *E*_a_ can be expressed: *E*_a_ = -R*k*ln10, where k is the slope of the fitting line, namely k is the (log*i*_*o*_)*/*(*1⁄T*). As shown in Fig. 7(c,d), the LiNi_0.03_Mg_0.08_Mn_1.89_O_4_ sample has a smaller *E*_a_ of 28.82 kJ·mol^−1^ than that of 30.77 kJ·mol^−1^ for the LiMg_0.08_Mn_1.92_O_4_. Therefore, the influence of Ni-Mg co-doping can effectively reduce the energy barrier by Li^+^ in the migration and diffusion. These results provide a convincing evidence for the enhanced rate capacity and cycling stability of the dual-doped LiNi_0.03_Mg_0.08_Mn_1.89_O_4_ electrode.

## Conclusions

In conclusion, we have successfully synthesized the LiNi_*x*_Mg_0.08_Mn_1.92−*x*_O_4_ (*x* ≤ 0.15) cathode materials by a facile solid-state combustion method. All as-prepared samples have pure spinel phase with polyhedron morphology. The cycling stability was enhanced both at 25 °C or elevated temperature, which was due to the robust structure stability. More importantly, the LiNi_0.03_Mg_0.08_Mn_1.89_O_4_ showed optimal electrochemical performance. And it delivered 104.0 mAh·g^−1^ at 5 C, however the LiMg_0.08_Mn_1.92_O_4_ was 94.7 mAh·g^−1^. Even at higher rate of 20 C, the LiNi_0.03_Mg_0.08_Mn_1.89_O_4_ remained excellent capacity retention of 75% after 1000 cycles, while the LiMg_0.08_Mn_1.92_O_4_ was only 56%. Such enhanced performances demonstrated that the addition of nickel ions into the Mg-doped spinel can remedy the shortcoming of the Mg-doping, and the Ni-Mg co-doping also improve the kinetic properties due to the large lithium ions diffusion coefficient of 1.359 × 10^−11^ cm^2^·s^−1^ and the small activation energy of 28.82 kJ·mol^−1^. Moreover, the structural stability of the LiMn_2_O_4_ is improved via the synergistic effect of nickel and magnesium ions. The as-prepared materials can draw widespread attention to the high performance lithium ion batteries.
